# (Methanol-κ*O*)(methano­lato-κ*O*)oxido[*N*-(2-oxidobenzyl­idene)isoleucinato-κ^3^ *O*,*N*,*O*′]vanadium(V)

**DOI:** 10.1107/S1600536812028231

**Published:** 2012-06-30

**Authors:** Chengyuan Wang, Zhenghua Guo, Jianfang Dong, Lianzhi Li

**Affiliations:** a Research Center of Medical Chemistry and Chemical Biology, Chongqing Technology and Business University, Chongqing 400067, People’s Republic of China; b School of Chemistry and Chemical Engineering, Liaocheng University, Shandong 252059, People’s Republic of China

## Abstract

In the title complex, [V(C_13_H_15_NO_3_)O(CH_3_O)(CH_3_OH)], the V^V^ atom is six-coordinated by a tridentate *O*,*N*,*O*′-donor ligand, derived from the condensation of salicyl­aldehyde and l-isoleucine, a vanadyl O atom, a methano­late O atom and a methanol O atom in a distorted octa­hedral geometry. The asymmetric unit contains two complex mol­ecules. In the crystal, inter­molecular O—H⋯O and C—H⋯O hydrogen bonds connect the mol­ecules into a one-dimensional chain along [100].

## Related literature

For background to vanadium compounds, see: Horn *et al.* (2004 ▶); Thompson *et al.* (1999 ▶); Wikksky *et al.* (2001 ▶). For related structures of vanadium complexes derived from amino acid Schiff base ligands and with a coordination number of six for vanadium, see: Bian & Li (2011 ▶); Cao *et al.* (2011 ▶); Chen *et al.* (2004 ▶).
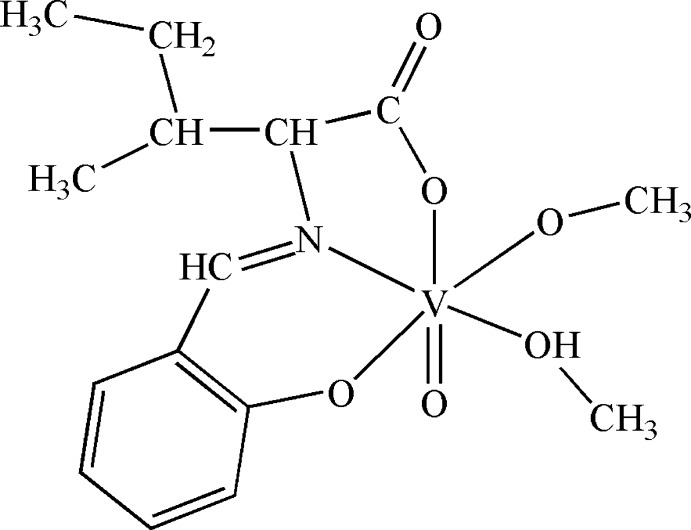


## Experimental

### 

#### Crystal data

[V(C_13_H_15_NO_3_)O(CH_3_O)(CH_4_O)]
*M* *_r_* = 363.28
Orthorhombic, 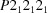

*a* = 6.6148 (9) Å
*b* = 18.463 (2) Å
*c* = 29.286 (3) Å
*V* = 3576.7 (7) Å^3^
*Z* = 8
Mo *K*α radiation
μ = 0.58 mm^−1^
*T* = 298 K
0.26 × 0.11 × 0.08 mm

#### Data collection

Bruker SMART 1000 CCD diffractometer
Absorption correction: multi-scan (*SADABS*; Sheldrick, 1996 ▶) *T*_min_ = 0.864, *T*_max_ = 0.955
18864 measured reflections
6295 independent reflections
3229 reflections with *I* > 2σ(*I*)
*R*_int_ = 0.174

#### Refinement

*R*[*F* ^2^ > 2σ(*F* ^2^)] = 0.092
*wR*(*F* ^2^) = 0.215
*S* = 1.04
6295 reflections
423 parameters
1046 restraints
H-atom parameters constrained
Δρ_max_ = 0.39 e Å^−3^
Δρ_min_ = −0.41 e Å^−3^
Absolute structure: Flack (1983 ▶), 2690 Friedel pairs
Flack parameter: 0.09 (5)

### 

Data collection: *SMART* (Bruker, 2007 ▶); cell refinement: *SAINT* (Bruker, 2007 ▶); data reduction: *SAINT*; program(s) used to solve structure: *SHELXS97* (Sheldrick, 2008 ▶); program(s) used to refine structure: *SHELXL97* (Sheldrick, 2008 ▶); molecular graphics: *XP* in *SHELXTL* (Sheldrick, 2008 ▶) and *DIAMOND* (Brandenburg, 1999 ▶); software used to prepare material for publication: *SHELXTL*.

## Supplementary Material

Crystal structure: contains datablock(s) global, I. DOI: 10.1107/S1600536812028231/hy2555sup1.cif

Structure factors: contains datablock(s) I. DOI: 10.1107/S1600536812028231/hy2555Isup2.hkl

Additional supplementary materials:  crystallographic information; 3D view; checkCIF report

## Figures and Tables

**Table 1:** Hydrogen-bond geometry (Å, °)

*D*—H⋯*A*	*D*—H	H⋯*A*	*D*⋯*A*	*D*—H⋯*A*
C2—H2⋯O5^i^	0.98	2.52	3.388 (12)	148
C7—H7⋯O4^i^	0.93	2.48	3.395 (12)	170
C17—H17⋯O12^i^	0.98	2.39	3.328 (12)	159
O6—H6⋯O8	0.82	1.89	2.688 (8)	165
O11—H11⋯O2	0.82	1.86	2.668 (8)	170

Symmetry code: (i) 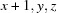.

**Fig. 1.**
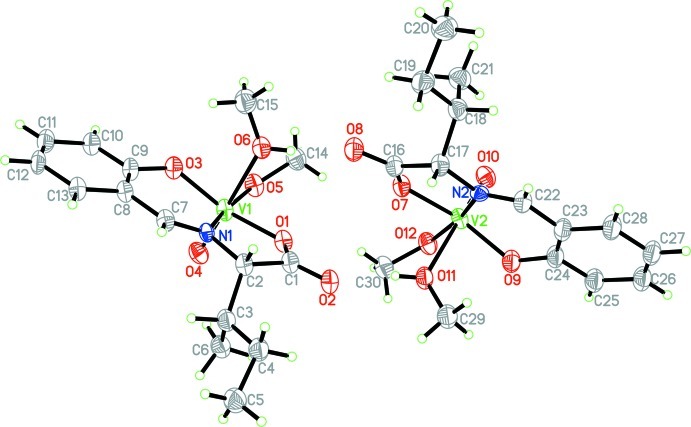
The molecular structure of the title compound, showing the 30% probability displacement ellipsoids.

**Fig. 2.**
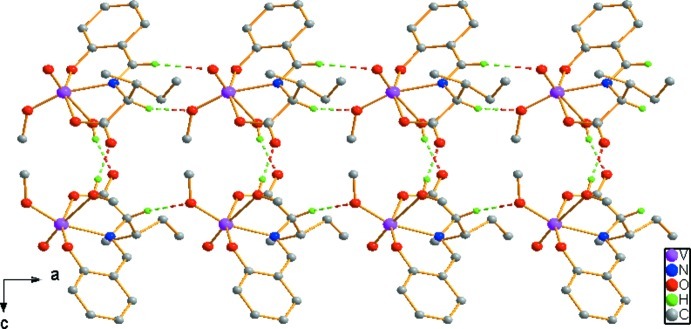
The one-dimensional structure of the title compound. Hydrogen bonds are shown as dashed lines.

## supplementary crystallographic information

### Comment

The strong interest in vanadium compounds arises from the presence of vanadium in several metalloenzymes, their use as metallopharmaceutical agents and their catalytic abilities (Horn *et al.*, 2004). Compared with other transition metal complexes, less vanadium complexes have been synthesized and characterized (Thompson *et al.*, 1999; Wikksky *et al.*, 2001). We report herein the synthesis and crystal structure of a new oxovanadium(V) complex with a tridentate Schiff base ligand derived from the condensation of salicylaldehyde and *L*-isoleucine.

As shown in Fig. 1, the asymmetric unit of the title compound contains two independent molecules. Each V^V^ ion is six-coordinated by a tridentate *O,N,O*-donor ligand, a vanadyl O atom, a methanolate O atom and a methanol O atom, forming a distorted octahedral geometry. In one of the complex molecules, O1, N1, O3 atoms of the Schiff base ligand and O5 atom of the methanolate define the equatorial plane and the terminal oxido O4 and the methanol O6 are at the axial positions. The V1 atom lies 0.308 (3) Å above the equatorial plane towards O4. The V2 atom deviates 0.297 (3) Å from the equatorial plane, formed by O7, N2, O9 and O12, towards O10. The axial O6 and O11 atoms are involved in long V—O distances [V1—O6 and V2—O11 = 2.345 (6) and 2.330 (6) Å], which is similar to the reported vanadium(V) complexes (Bian & Li, 2011; Cao *et al.*, 2011; Chen *et al.*, 2004). In the crystal, intermolecular O—H···O and C—H···O hydrogen bonds connect the molecules into a one-dimensional structure along [100] (Table 1, Fig. 2).

### Experimental

*L*-Isoleucine (1 mmol, 131.2 mg) and potassium hydroxide (1 mmol, 56.1 mg) were dissolved in hot methanol (10 ml) with stirring and added successively to a methanol solution (5 ml) of salicylaldehyde (1 mmol, 0.11 ml). The mixture was then stirred at 333 K for 2 h. Subsequently, an aqueous solution (2 ml) of vanadyl sulfate hydrate (1 mmol, 225.4 mg) was added dropwise and stirred for 2 h continuously. Then the resultant solution was filtered and the filtrate was held at room temperature for several days, whereupon brown blocky crystals suitable for X-ray diffraction were obtained.

### Refinement

H atoms were positioned geometrically and refined as riding atoms, with C—H = 0.93 (aromatic), 0.98 (CH), 0.97 (CH_2_) and 0.96 (CH_3_) Å and O—H = 0.82 Å and with *U*_iso_(H) = 1.2(1.5 for methyl and hydroxyl)*U*_eq_(C, O).

### Crystal data

**Table:**

[V(C_13_H_15_NO_3_)O(CH_3_O)(CH_4_O)]	*F*(000) = 1520
*M**_r_* = 363.28	*D*_x_ = 1.349 Mg m^−^^3^
Orthorhombic, *P*2_1_2_1_2_1_	Mo *K*α radiation, λ = 0.71073 Å
Hall symbol: P 2ac 2ab	Cell parameters from 2166 reflections
*a* = 6.6148 (9) Å	θ = 2.6–25.2°
*b* = 18.463 (2) Å	µ = 0.58 mm^−^^1^
*c* = 29.286 (3) Å	*T* = 298 K
*V* = 3576.7 (7) Å^3^	Block, brown
*Z* = 8	0.26 × 0.11 × 0.08 mm

### Data collection

**Table:**

Bruker SMART 1000 CCD diffractometer	6295 independent reflections
Radiation source: fine-focus sealed tube	3229 reflections with *I* > 2σ(*I*)
Graphite monochromator	*R*_int_ = 0.174
φ and ω scans	θ_max_ = 25.0°, θ_min_ = 1.3°
Absorption correction: multi-scan (*SADABS*; Sheldrick, 1996)	*h* = −7→7
*T*_min_ = 0.864, *T*_max_ = 0.955	*k* = −12→21
18864 measured reflections	*l* = −34→34

### Refinement

**Table:**

Refinement on *F*^2^	Secondary atom site location: difference Fourier map
Least-squares matrix: full	Hydrogen site location: inferred from neighbouring sites
*R*[*F*^2^ > 2σ(*F*^2^)] = 0.092	H-atom parameters constrained
*wR*(*F*^2^) = 0.215	*w* = 1/[σ^2^(*F*_o_^2^) + (0.0752*P*)^2^] where *P* = (*F*_o_^2^ + 2*F*_c_^2^)/3
*S* = 1.04	(Δ/σ)_max_ = 0.007
6295 reflections	Δρ_max_ = 0.39 e Å^−^^3^
423 parameters	Δρ_min_ = −0.41 e Å^−^^3^
1046 restraints	Absolute structure: Flack (1983), 2690 Friedel pairs
Primary atom site location: structure-invariant direct methods	Flack parameter: 0.09 (5)

### Special details

**Table:**

Geometry. All e.s.d.'s (except the e.s.d. in the dihedral angle between two l.s. planes) are estimated using the full covariance matrix. The cell e.s.d.'s are taken into account individually in the estimation of e.s.d.'s in distances, angles and torsion angles; correlations between e.s.d.'s in cell parameters are only used when they are defined by crystal symmetry. An approximate (isotropic) treatment of cell e.s.d.'s is used for estimating e.s.d.'s involving l.s. planes.
Refinement. Refinement of *F*^2^ against ALL reflections. The weighted *R*-factor *wR* and goodness of fit *S* are based on *F*^2^, conventional *R*-factors *R* are based on *F*, with *F* set to zero for negative *F*^2^. The threshold expression of *F*^2^ > σ(*F*^2^) is used only for calculating *R*-factors(gt) *etc*. and is not relevant to the choice of reflections for refinement. *R*-factors based on *F*^2^ are statistically about twice as large as those based on *F*, and *R*- factors based on ALL data will be even larger.

### Fractional atomic coordinates and isotropic or equivalent isotropic displacement parameters (Å^2^)

**Table:**

	*x*	*y*	*z*	*U*_iso_*/*U*_eq_	
V1	0.6186 (2)	0.58076 (9)	0.08740 (5)	0.0456 (4)	
V2	0.6024 (2)	0.68203 (8)	0.30480 (5)	0.0455 (4)	
N1	0.9207 (11)	0.6096 (4)	0.0724 (2)	0.0360 (17)	
N2	0.8986 (11)	0.6526 (3)	0.3246 (2)	0.0369 (16)	
O1	0.6870 (9)	0.6492 (3)	0.1357 (2)	0.0532 (12)	
O2	0.9033 (11)	0.7236 (3)	0.17086 (19)	0.0581 (12)	
O3	0.6656 (9)	0.4999 (3)	0.05076 (19)	0.0554 (13)	
O4	0.5227 (10)	0.6343 (3)	0.0500 (2)	0.0575 (14)	
O5	0.4003 (11)	0.5519 (3)	0.11735 (19)	0.0573 (14)	
O6	0.7934 (9)	0.5063 (3)	0.1388 (2)	0.0545 (13)	
H6	0.8105	0.5218	0.1648	0.065*	
O7	0.6786 (9)	0.6122 (3)	0.25799 (19)	0.0526 (12)	
O8	0.9174 (10)	0.5451 (3)	0.2227 (2)	0.0605 (13)	
O9	0.6394 (11)	0.7641 (3)	0.34196 (19)	0.0596 (13)	
O10	0.4947 (10)	0.6297 (3)	0.3407 (2)	0.0575 (14)	
O11	0.7984 (9)	0.7540 (3)	0.25673 (19)	0.0547 (13)	
H11	0.8258	0.7399	0.2309	0.066*	
O12	0.3896 (8)	0.7086 (3)	0.27030 (18)	0.0568 (13)	
C1	0.8593 (16)	0.6819 (5)	0.1394 (3)	0.0525 (15)	
C2	1.0115 (14)	0.6683 (5)	0.1006 (3)	0.0488 (15)	
H2	1.1374	0.6501	0.1140	0.059*	
C3	1.0599 (15)	0.7389 (5)	0.0734 (3)	0.0545 (17)	
H3	1.1291	0.7232	0.0455	0.065*	
C4	1.2096 (16)	0.7875 (5)	0.0984 (3)	0.0583 (19)	
H4A	1.1409	0.8101	0.1240	0.070*	
H4B	1.3172	0.7576	0.1107	0.070*	
C5	1.3008 (16)	0.8451 (5)	0.0698 (3)	0.068 (2)	
H5A	1.3959	0.8239	0.0489	0.103*	
H5B	1.3691	0.8795	0.0890	0.103*	
H5C	1.1964	0.8693	0.0528	0.103*	
C6	0.8720 (17)	0.7769 (5)	0.0578 (3)	0.0608 (17)	
H6A	0.8152	0.8037	0.0828	0.091*	
H6B	0.7757	0.7418	0.0472	0.091*	
H6C	0.9048	0.8095	0.0334	0.091*	
C7	1.0345 (15)	0.5792 (5)	0.0418 (3)	0.0517 (15)	
H7	1.1677	0.5950	0.0400	0.062*	
C8	0.9732 (15)	0.5228 (5)	0.0103 (3)	0.0486 (16)	
C9	0.7908 (15)	0.4889 (5)	0.0143 (3)	0.0534 (16)	
C10	0.7324 (17)	0.4356 (5)	−0.0177 (3)	0.0582 (18)	
H10	0.6086	0.4120	−0.0153	0.070*	
C11	0.8681 (17)	0.4195 (5)	−0.0533 (3)	0.0600 (18)	
H11A	0.8332	0.3845	−0.0746	0.072*	
C12	1.0428 (15)	0.4528 (5)	−0.0570 (3)	0.0548 (18)	
H12A	1.1263	0.4411	−0.0815	0.066*	
C13	1.1091 (17)	0.5055 (5)	−0.0256 (3)	0.0549 (17)	
H13	1.2348	0.5277	−0.0282	0.066*	
C14	0.3652 (17)	0.5267 (5)	0.1638 (3)	0.066 (2)	
H14A	0.3936	0.5653	0.1848	0.099*	
H14B	0.4521	0.4863	0.1701	0.099*	
H14C	0.2267	0.5121	0.1670	0.099*	
C15	0.8635 (18)	0.4359 (5)	0.1361 (3)	0.073 (2)	
H15A	0.9531	0.4265	0.1612	0.109*	
H15B	0.9349	0.4294	0.1079	0.109*	
H15C	0.7513	0.4030	0.1374	0.109*	
C16	0.8581 (16)	0.5828 (5)	0.2552 (3)	0.0513 (14)	
C17	1.0050 (15)	0.5991 (5)	0.2949 (3)	0.0518 (15)	
H17	1.1290	0.6209	0.2828	0.062*	
C18	1.0586 (14)	0.5289 (5)	0.3221 (3)	0.0563 (17)	
H18	1.1187	0.5441	0.3512	0.068*	
C19	1.2219 (16)	0.4839 (5)	0.2959 (3)	0.0633 (19)	
H19A	1.1597	0.4628	0.2690	0.076*	
H19B	1.3272	0.5166	0.2856	0.076*	
C20	1.3212 (16)	0.4223 (5)	0.3243 (4)	0.074 (2)	
H20A	1.3860	0.4425	0.3507	0.111*	
H20B	1.4197	0.3977	0.3058	0.111*	
H20C	1.2191	0.3885	0.3338	0.111*	
C21	0.8721 (17)	0.4819 (5)	0.3335 (3)	0.0662 (17)	
H21A	0.8137	0.4639	0.3058	0.099*	
H21B	0.7742	0.5108	0.3495	0.099*	
H21C	0.9126	0.4420	0.3524	0.099*	
C22	1.0021 (16)	0.6793 (5)	0.3590 (3)	0.0547 (15)	
H22	1.1333	0.6629	0.3639	0.066*	
C23	0.9192 (17)	0.7337 (5)	0.3899 (3)	0.0560 (16)	
C24	0.7400 (17)	0.7719 (5)	0.3813 (3)	0.0570 (16)	
C25	0.6718 (17)	0.8226 (6)	0.4131 (3)	0.0658 (18)	
H25	0.5537	0.8485	0.4076	0.079*	
C26	0.7817 (17)	0.8347 (6)	0.4538 (3)	0.0662 (19)	
H26	0.7359	0.8688	0.4748	0.079*	
C27	0.9519 (17)	0.7975 (5)	0.4623 (3)	0.064 (2)	
H27	1.0196	0.8051	0.4897	0.077*	
C28	1.0326 (17)	0.7461 (6)	0.4303 (3)	0.0658 (19)	
H28	1.1537	0.7220	0.4357	0.079*	
C29	0.9195 (16)	0.8146 (5)	0.2678 (3)	0.0646 (19)	
H29A	0.8386	0.8502	0.2832	0.097*	
H29B	0.9736	0.8352	0.2403	0.097*	
H29C	1.0284	0.7997	0.2873	0.097*	
C30	0.3799 (17)	0.7283 (5)	0.2223 (3)	0.0633 (19)	
H30A	0.2421	0.7263	0.2121	0.095*	
H30B	0.4601	0.6951	0.2047	0.095*	
H30C	0.4310	0.7765	0.2183	0.095*	

### Atomic displacement parameters (Å^2^)

**Table:**

	*U*^11^	*U*^22^	*U*^33^	*U*^12^	*U*^13^	*U*^23^
V1	0.0333 (8)	0.0662 (10)	0.0373 (8)	−0.0008 (9)	−0.0012 (8)	−0.0034 (8)
V2	0.0409 (9)	0.0609 (10)	0.0346 (8)	−0.0015 (9)	0.0004 (8)	0.0037 (8)
N1	0.036 (4)	0.046 (4)	0.026 (3)	0.000 (4)	−0.008 (3)	−0.001 (3)
N2	0.037 (4)	0.032 (3)	0.042 (4)	0.009 (4)	0.004 (4)	0.003 (3)
O1	0.051 (2)	0.067 (2)	0.041 (2)	0.002 (2)	0.000 (2)	−0.002 (2)
O2	0.064 (3)	0.071 (2)	0.040 (2)	0.000 (2)	0.004 (2)	−0.008 (2)
O3	0.055 (3)	0.067 (3)	0.044 (2)	−0.005 (2)	0.002 (2)	−0.007 (2)
O4	0.053 (3)	0.073 (3)	0.046 (3)	−0.005 (2)	−0.007 (2)	0.008 (2)
O5	0.044 (3)	0.078 (3)	0.050 (3)	−0.006 (3)	−0.002 (3)	0.000 (3)
O6	0.058 (3)	0.063 (2)	0.043 (2)	0.002 (2)	−0.001 (2)	−0.002 (2)
O7	0.052 (2)	0.065 (2)	0.040 (2)	0.005 (2)	−0.005 (2)	−0.003 (2)
O8	0.065 (3)	0.070 (3)	0.047 (2)	0.010 (3)	−0.004 (2)	−0.008 (2)
O9	0.067 (3)	0.066 (3)	0.045 (2)	0.002 (3)	−0.001 (2)	−0.007 (2)
O10	0.053 (3)	0.071 (3)	0.048 (3)	0.002 (3)	0.007 (2)	0.006 (2)
O11	0.062 (3)	0.064 (3)	0.038 (2)	−0.006 (2)	0.003 (2)	−0.005 (2)
O12	0.047 (3)	0.077 (3)	0.046 (3)	0.001 (3)	−0.004 (2)	0.005 (3)
C1	0.057 (3)	0.062 (3)	0.038 (3)	−0.001 (3)	−0.002 (3)	−0.002 (3)
C2	0.049 (3)	0.060 (3)	0.037 (3)	−0.002 (3)	0.001 (3)	−0.001 (3)
C3	0.060 (3)	0.062 (3)	0.041 (3)	−0.004 (3)	0.007 (3)	−0.002 (3)
C4	0.066 (4)	0.062 (4)	0.047 (4)	−0.007 (3)	0.007 (3)	−0.003 (3)
C5	0.075 (5)	0.073 (5)	0.057 (4)	−0.018 (4)	0.006 (4)	−0.003 (4)
C6	0.064 (3)	0.070 (3)	0.049 (3)	−0.005 (3)	0.006 (3)	0.002 (3)
C7	0.054 (3)	0.061 (3)	0.039 (3)	0.003 (3)	0.001 (3)	−0.001 (3)
C8	0.053 (3)	0.060 (3)	0.033 (3)	−0.001 (3)	0.003 (3)	−0.002 (3)
C9	0.058 (3)	0.066 (3)	0.035 (3)	0.000 (3)	0.001 (3)	−0.006 (3)
C10	0.064 (4)	0.067 (4)	0.043 (3)	−0.001 (3)	0.000 (3)	−0.006 (3)
C11	0.070 (4)	0.068 (4)	0.042 (3)	0.003 (4)	0.000 (3)	−0.011 (3)
C12	0.067 (4)	0.066 (4)	0.031 (3)	0.006 (3)	0.003 (3)	−0.013 (3)
C13	0.060 (3)	0.066 (3)	0.039 (3)	0.002 (3)	0.005 (3)	−0.005 (3)
C14	0.060 (4)	0.082 (4)	0.056 (4)	−0.001 (4)	0.011 (4)	0.005 (4)
C15	0.076 (4)	0.082 (4)	0.061 (4)	0.012 (4)	−0.004 (4)	−0.003 (4)
C16	0.056 (3)	0.059 (3)	0.039 (3)	0.004 (3)	−0.001 (3)	−0.002 (3)
C17	0.053 (3)	0.060 (3)	0.043 (3)	0.003 (3)	−0.003 (3)	−0.004 (3)
C18	0.059 (3)	0.063 (3)	0.047 (3)	0.006 (3)	−0.009 (3)	−0.002 (3)
C19	0.065 (4)	0.062 (4)	0.063 (4)	0.009 (3)	−0.006 (3)	−0.004 (3)
C20	0.074 (5)	0.069 (5)	0.079 (5)	0.004 (4)	−0.010 (4)	0.000 (4)
C21	0.068 (3)	0.069 (3)	0.062 (3)	0.009 (3)	−0.002 (3)	0.005 (3)
C22	0.060 (3)	0.060 (3)	0.044 (3)	0.000 (3)	−0.002 (3)	−0.007 (3)
C23	0.064 (3)	0.062 (3)	0.042 (3)	−0.006 (3)	−0.002 (3)	−0.010 (3)
C24	0.067 (3)	0.065 (3)	0.039 (3)	−0.003 (3)	0.005 (3)	−0.007 (3)
C25	0.076 (4)	0.073 (4)	0.048 (3)	0.000 (3)	0.005 (3)	−0.010 (3)
C26	0.077 (4)	0.074 (4)	0.047 (3)	−0.003 (4)	0.004 (3)	−0.013 (4)
C27	0.079 (4)	0.069 (4)	0.045 (3)	−0.006 (4)	0.000 (3)	−0.015 (3)
C28	0.077 (4)	0.072 (4)	0.048 (3)	0.000 (3)	−0.003 (3)	−0.013 (3)
C29	0.071 (4)	0.072 (4)	0.051 (4)	−0.007 (4)	0.001 (4)	−0.002 (4)
C30	0.060 (4)	0.079 (4)	0.050 (4)	0.007 (4)	−0.002 (4)	0.003 (3)

### Geometric parameters (Å, º)

**Table:**

V1—O4	1.606 (6)	C9—C10	1.413 (12)
V1—O5	1.772 (7)	C10—C11	1.407 (13)
V1—O3	1.865 (6)	C10—H10	0.9300
V1—O1	1.950 (6)	C11—C12	1.313 (13)
V1—N1	2.114 (7)	C11—H11A	0.9300
V1—O6	2.345 (6)	C12—C13	1.410 (12)
V2—O10	1.596 (6)	C12—H12A	0.9300
V2—O12	1.801 (4)	C13—H13	0.9300
V2—O9	1.881 (6)	C14—H14A	0.9600
V2—O7	1.948 (6)	C14—H14B	0.9600
V2—N2	2.114 (8)	C14—H14C	0.9600
V2—O11	2.330 (6)	C15—H15A	0.9600
N1—C7	1.298 (10)	C15—H15B	0.9600
N1—C2	1.490 (10)	C15—H15C	0.9600
N2—C22	1.314 (11)	C16—C17	1.543 (12)
N2—C17	1.492 (10)	C17—C18	1.564 (12)
O1—C1	1.294 (11)	C17—H17	0.9800
O2—C1	1.236 (10)	C18—C19	1.564 (12)
O3—C9	1.366 (10)	C18—C21	1.544 (14)
O5—C14	1.455 (10)	C18—H18	0.9800
O6—C15	1.382 (10)	C19—C20	1.555 (12)
O6—H6	0.8200	C19—H19A	0.9700
O7—C16	1.308 (11)	C19—H19B	0.9700
O8—C16	1.242 (9)	C20—H20A	0.9600
O9—C24	1.339 (11)	C20—H20B	0.9600
O11—C29	1.414 (10)	C20—H20C	0.9600
O11—H11	0.8200	C21—H21A	0.9600
O12—C30	1.454 (9)	C21—H21B	0.9600
C1—C2	1.538 (12)	C21—H21C	0.9600
C2—C3	1.562 (12)	C22—C23	1.459 (12)
C2—H2	0.9800	C22—H22	0.9300
C3—C6	1.498 (13)	C23—C24	1.402 (14)
C3—C4	1.524 (12)	C23—C28	1.418 (13)
C3—H3	0.9800	C24—C25	1.396 (13)
C4—C5	1.483 (12)	C25—C26	1.413 (13)
C4—H4A	0.9700	C25—H25	0.9300
C4—H4B	0.9700	C26—C27	1.343 (14)
C5—H5A	0.9600	C26—H26	0.9300
C5—H5B	0.9600	C27—C28	1.437 (13)
C5—H5C	0.9600	C27—H27	0.9300
C6—H6A	0.9600	C28—H28	0.9300
C6—H6B	0.9600	C29—H29A	0.9600
C6—H6C	0.9600	C29—H29B	0.9600
C7—C8	1.449 (12)	C29—H29C	0.9600
C7—H7	0.9300	C30—H30A	0.9600
C8—C9	1.364 (12)	C30—H30B	0.9600
C8—C13	1.419 (12)	C30—H30C	0.9600
			
O4—V1—O5	101.6 (3)	C9—C10—C11	117.6 (10)
O4—V1—O3	99.6 (3)	C9—C10—H10	121.2
O5—V1—O3	100.3 (3)	C11—C10—H10	121.2
O4—V1—O1	100.8 (3)	C12—C11—C10	121.6 (9)
O5—V1—O1	91.5 (3)	C12—C11—H11A	119.2
O3—V1—O1	153.8 (3)	C10—C11—H11A	119.2
O4—V1—N1	94.4 (3)	C11—C12—C13	122.8 (9)
O5—V1—N1	161.7 (3)	C11—C12—H12A	118.6
O3—V1—N1	85.7 (3)	C13—C12—H12A	118.6
O1—V1—N1	76.6 (2)	C8—C13—C12	116.2 (10)
O4—V1—O6	173.7 (3)	C8—C13—H13	121.9
O5—V1—O6	84.7 (3)	C12—C13—H13	121.9
O3—V1—O6	79.5 (2)	O5—C14—H14A	109.5
O1—V1—O6	78.4 (2)	O5—C14—H14B	109.5
N1—V1—O6	79.4 (2)	H14A—C14—H14B	109.5
O10—V2—O12	100.7 (3)	O5—C14—H14C	109.5
O10—V2—O9	99.4 (3)	H14A—C14—H14C	109.5
O12—V2—O9	101.9 (3)	H14B—C14—H14C	109.5
O10—V2—O7	100.3 (3)	O6—C15—H15A	109.5
O12—V2—O7	89.3 (3)	O6—C15—H15B	109.5
O9—V2—O7	155.0 (3)	H15A—C15—H15B	109.5
O10—V2—N2	94.4 (3)	O6—C15—H15C	109.5
O12—V2—N2	161.5 (3)	H15A—C15—H15C	109.5
O9—V2—N2	85.9 (3)	H15B—C15—H15C	109.5
O7—V2—N2	77.5 (3)	O8—C16—O7	124.5 (9)
O10—V2—O11	172.7 (3)	O8—C16—C17	119.1 (9)
O12—V2—O11	86.6 (3)	O7—C16—C17	116.3 (8)
O9—V2—O11	79.5 (2)	N2—C17—C16	105.7 (7)
O7—V2—O11	78.9 (2)	N2—C17—C18	110.9 (7)
N2—V2—O11	78.3 (2)	C16—C17—C18	111.4 (7)
C7—N1—C2	117.7 (7)	N2—C17—H17	109.6
C7—N1—V1	125.6 (6)	C16—C17—H17	109.6
C2—N1—V1	116.7 (5)	C18—C17—H17	109.6
C22—N2—C17	116.7 (8)	C19—C18—C21	111.1 (7)
C22—N2—V2	126.6 (6)	C19—C18—C17	110.3 (8)
C17—N2—V2	116.5 (5)	C21—C18—C17	113.3 (8)
C1—O1—V1	124.6 (6)	C19—C18—H18	107.3
C9—O3—V1	132.0 (6)	C21—C18—H18	107.3
C14—O5—V1	133.5 (6)	C17—C18—H18	107.3
C15—O6—V1	132.8 (6)	C20—C19—C18	114.8 (8)
C15—O6—H6	109.6	C20—C19—H19A	108.6
V1—O6—H6	117.4	C18—C19—H19A	108.6
C16—O7—V2	123.6 (6)	C20—C19—H19B	108.6
C24—O9—V2	130.5 (6)	C18—C19—H19B	108.6
C29—O11—V2	128.9 (5)	H19A—C19—H19B	107.6
C29—O11—H11	109.7	C19—C20—H20A	109.5
V2—O11—H11	119.9	C19—C20—H20B	109.5
C30—O12—V2	130.2 (6)	H20A—C20—H20B	109.5
O2—C1—O1	124.1 (9)	C19—C20—H20C	109.5
O2—C1—C2	119.9 (9)	H20A—C20—H20C	109.5
O1—C1—C2	116.0 (8)	H20B—C20—H20C	109.5
N1—C2—C1	105.3 (7)	C18—C21—H21A	109.5
N1—C2—C3	114.0 (7)	C18—C21—H21B	109.5
C1—C2—C3	112.1 (7)	H21A—C21—H21B	109.5
N1—C2—H2	108.4	C18—C21—H21C	109.5
C1—C2—H2	108.4	H21A—C21—H21C	109.5
C3—C2—H2	108.4	H21B—C21—H21C	109.5
C6—C3—C4	114.2 (8)	N2—C22—C23	122.6 (9)
C6—C3—C2	112.1 (8)	N2—C22—H22	118.7
C4—C3—C2	112.3 (7)	C23—C22—H22	118.7
C6—C3—H3	105.8	C24—C23—C28	121.0 (9)
C4—C3—H3	105.8	C24—C23—C22	123.6 (9)
C2—C3—H3	105.8	C28—C23—C22	115.4 (9)
C5—C4—C3	114.5 (8)	O9—C24—C25	119.1 (10)
C5—C4—H4A	108.6	O9—C24—C23	121.4 (9)
C3—C4—H4A	108.6	C25—C24—C23	119.4 (10)
C5—C4—H4B	108.6	C24—C25—C26	120.2 (11)
C3—C4—H4B	108.6	C24—C25—H25	119.9
H4A—C4—H4B	107.6	C26—C25—H25	119.9
C4—C5—H5A	109.5	C27—C26—C25	120.4 (10)
C4—C5—H5B	109.5	C27—C26—H26	119.8
H5A—C5—H5B	109.5	C25—C26—H26	119.8
C4—C5—H5C	109.5	C26—C27—C28	121.9 (10)
H5A—C5—H5C	109.5	C26—C27—H27	119.0
H5B—C5—H5C	109.5	C28—C27—H27	119.0
C3—C6—H6A	109.5	C23—C28—C27	117.0 (10)
C3—C6—H6B	109.5	C23—C28—H28	121.5
H6A—C6—H6B	109.5	C27—C28—H28	121.5
C3—C6—H6C	109.5	O11—C29—H29A	109.5
H6A—C6—H6C	109.5	O11—C29—H29B	109.5
H6B—C6—H6C	109.5	H29A—C29—H29B	109.5
N1—C7—C8	126.0 (9)	O11—C29—H29C	109.5
N1—C7—H7	117.0	H29A—C29—H29C	109.5
C8—C7—H7	117.0	H29B—C29—H29C	109.5
C9—C8—C13	121.4 (9)	O12—C30—H30A	109.5
C9—C8—C7	121.4 (9)	O12—C30—H30B	109.5
C13—C8—C7	117.1 (9)	H30A—C30—H30B	109.5
O3—C9—C8	122.4 (8)	O12—C30—H30C	109.5
O3—C9—C10	117.1 (9)	H30A—C30—H30C	109.5
C8—C9—C10	120.3 (9)	H30B—C30—H30C	109.5
			
C1—C2—C3—C4	78.7 (10)		

### Hydrogen-bond geometry (Å, º)

**Table:**

*D*—H···*A*	*D*—H	H···*A*	*D*···*A*	*D*—H···*A*
C2—H2···O5^i^	0.98	2.52	3.388 (12)	148
C7—H7···O4^i^	0.93	2.48	3.395 (12)	170
C17—H17···O12^i^	0.98	2.39	3.328 (12)	159
O6—H6···O8	0.82	1.89	2.688 (8)	165
O11—H11···O2	0.82	1.86	2.668 (8)	170

Symmetry code: (i) *x*+1, *y*, *z*.
